# The use of reflective diaries in end of life training programmes: a study exploring the impact of self-reflection on the participants in a volunteer training programme

**DOI:** 10.1186/s12904-016-0096-5

**Published:** 2016-03-05

**Authors:** Alison Germain, Kate Nolan, Rita Doyle, Stephen Mason, Maureen Gambles, Hong Chen, Ruthmarijke Smeding, John Ellershaw

**Affiliations:** Department of Molecular & Clinical Cancer Medicine, Marie Curie Palliative Care Institute Liverpool, University of Liverpool, Cancer Research Centre, 200 London Road, Liverpool, L3 9TA UK

**Keywords:** International, Education and training, Dying, Volunteers, Reflective diaries, Personal growth, Bereavement

## Abstract

**Background:**

A training programme was developed and delivered to a cohort of volunteers who were preparing for a unique role to provide companionship to dying patients in the acute hospital setting. This comprehensive programme aimed to provide an opportunity for participants to fully understand the nature and responsibilities of the role, whilst also allowing sufficient time to assess the qualities and competencies of participants for their ongoing volunteering role.

Participants completed reflective diaries throughout the training course to record their ongoing thoughts and feelings. The purpose of this paper is to present a phenomenological analysis of these entries to understand participants’ experiences, perceptions and motivations.

**Method:**

The wider study was structured into three phases. Phase 1 was the delivery of a 12 week, bespoke training programme; Phase 2 involved a 26 week pilot implementation of the Care of the Dying Volunteer Service and Phase 3 was the research evaluation of the training and implementation which would inform the further development of the training programme.

Self-reflection is a common component of End of Life training programmes and volunteers in this study completed a reflective diary after participation in each of the training sessions. A thematic analysis was undertaken to explore and understand the participants’ experience, perceptions and motivations in relation to their participation in the training.

**Results:**

All 19 volunteers completed the reflective diaries. From a potential 228 diary entries over the 12 week training programme, 178 diary entries were submitted (78 %). The following key themes were identified: Dying Alone and the importance of being present, Personal loss and the reconstruction of meaning, Self-Awareness and Personal growth, Self-preservation and Coping strategies and group unity/cohesion.

**Conclusions:**

The participants in this study demonstrated that they were able to use the diaries as an appropriate medium for reflection. Their reflections were also instrumental in the ongoing revision and development of the training programme. Analysis of their entries illustrated that the diaries could provide the opportunity for a reappraisal of their world view and personal philosophy around death and dying. Further research is undoubtedly required, however this paper suggests that self-reflection in this way, supports preparation in honing the appropriate attitudes and qualities required to work in this role.

## Background

In this study an Education and Training programme was developed and delivered to a cohort of volunteers. The participants were preparing for a unique and innovative role to provide a presence or companionship to dying patients in the last hours and days of life in an acute hospital. The volunteer role was created to provide a support to imminently dying patients who had few or no visitors, or conversely, where visitors at the bedside were in need of respite from their ‘vigil’. The training programme was developed from the work of OPCARE 9, an EU funded 7th Framework project that included a focus on exploring the role of the Volunteer in end of life care in nine European countries [1]. The aim of this element of the work in OPCARE9 was to understand current volunteer education and training provision, specifically related to the last weeks and days of life, and to identify any gaps in training, in order to establish curricula excellence in this area. The paucity of examples of the use of volunteers in the UK at this specific time, led to the development of a core curriculum for training in the current project. It sought to distil international expertise and experience to underpin and deliver a bespoke volunteering model to provide support at the bedside of dying patients in one acute hospital in the UK.

After this initial development, the remainder of the study was structured into three phases. Phase 1 was the delivery of a 12 week, training programme; Phase 2 involved a 26 week pilot implementation of the Care of the Dying Volunteer Service and Phase 3 was the research evaluation of the training and implementation phases (See Table 1).

**Table 1:** Project structure

Project structure		
Phase	Objectives	Description
Phase 1	Design of education and training programme	To: • provide adequate preparation for the volunteering role • hone existing skills • develop awareness of end of life care • explore individual’s sense of resilience and ability to cope with the demands of the role • avoid professionalization of the role Three full days 9 × 2 h sessions Shadowing Hospital Specialist Palliative Care Team Five modules: • Process of death and dying • Emotional support • Spiritual support • Communication skills • Understanding the CODV role and boundaries
	Delivery of 12 week education and training programme	
Phase 2	26 week pilot implementation	• Six wards purposively chosen within the Trust for their high proportion of expected deaths and referrals to specialist palliative care • Ward areas prepared - dissemination of study/service information via briefing with healthcare professionals • Referral criteria – patients believed to be in the last hours or days of life who had been referred to the Hospital Specialist Palliative Care Team • Service offered to patients who had no or few visitors and to the families and friends of patients who were in need of a break from their bedside vigil.
Phase 3	Research evaluation of the CODV programme and implementation	• mixed methods evaluation employing questionnaires and phenomenological interviews • All nurses and healthcare assistants on the pilot wards invited to complete a study specific questionnaire to assess attitudes to and expectations of volunteers in care of the dying prior to implementation • Phenomenological interviews undertaken with volunteers, staff and bereaved relatives who had been directly involved in a support episode during the pilot phase, to gain an understanding of their experiences and views of the service • Questionnaire data analysed descriptively (means, medians, proportions, as appropriate) • Interview data transcribed verbatim and thematically analysed.

The comprehensive Education and Training programme aimed at providing an appropriate understanding and preparation participant’s ongoing role, whilst also allowing the Training Leads sufficient time to assess their qualities and competencies. Clearly, the sensitivity of the role, supporting dying patients and their families, demanded a thorough preparation which included a personal reflection of previous losses and bereavements and an exposure to the visual images of death that they might be faced with in their uptake of this role. The learning objectives were designed to balance the functional requirements of the volunteering role. This included an awareness of end of life care, a honing of their communication skills and an exploration of their sense of resilience and ability to cope, whilst also ensuring that the training did not professionalise the role, compromising the value of the volunteers as lay, community representatives. Reflection was regarded as an essential component to both the training and the ongoing Volunteering role, facilitating both an increased personal awareness and an opportunity for the Facilitators to evaluate the impact and effectiveness of the training programme.

Self-reflection is a common component of many End of Life training programmes for both Volunteers and Health Care professionals, particularly as a means of identifying any potential barriers to communicating about death and dying [2]. Reflection is theorised by Boud et al. [3] to include the intellectual and affective activities that individuals engage in to explore their experiences and process knowledge, leading to new awareness or understanding. Reflection can also be a means of addressing any personal discomfort or emotion in supporting dying patients that may be rooted in past experience, culture, beliefs or inexperience. In essence, reflection is a method of reviewing past experiences, both personal and professional, examining their impact and outcomes, then applying these new insights or learning to future practice.

Sandars [4] states that the method of reflection which is used should be determined by the learning styles and preferences of the participants and the learning outcomes for the particular teaching programme. Previous studies have highlighted that for reflective learning to be effective, the process should be taught, with the facilitators assisting students in their assessment and analysis [4]. Without this level of guidance and challenge reflection may limit the opportunity for students to develop and expand their learning. In support, Ghaye [5] suggests that effective reflection should be organised and structured and triggered by a question which demands the participant to look critically at their actions and question their efficacy. Although there are other methods to promote reflection, Burrows [6] suggests that the simplicity of completing a reflective diary offers the most effective medium to those with little or no previous experience of reflective practice and provides a straightforward format to channel the process of reflective thinking.

The diaries also facilitated the formal documentation of the participants’ reflections and this gave access to a source of ‘rich data’ which according to Charmaz [7] can reveal participants’ thoughts and feelings that may not have been offered in other circumstances, such as through social interactions. Such ‘rich data’ can then be subjected to in-depth analysis in order to understand more broadly participants’ experiences, perceptions, motivations and actions. A particularly relevant research methodology is phenomenological inquiry, which with its emphasis on the importance of personal knowledge and interpretation, is a powerful tool that can be used to gain insights into lived experience of those who participate in the phenomenon under study [8].

The aim of this paper is to present the results of a phenomenological analysis of the diary entries that was undertaken to explore and understand the participants’ experience, perceptions and motivations in relation to the phenomenon under study, their participation in the CODV training.

## Methods

### Sample

The CODV programme was broadly advertised locally and initially 43 enquiries were received which resulted in 22 participants attending an interview. Three potential participants were excluded from the training at this point for the following reasons: one had experienced a recent bereavement; two could not commit fully to the 12 week training programme.

Reference and standard agency checks (Disclosing and Barring Services, formally Criminal Records Bureau) were conducted for the remaining 19 volunteers who attended the training programme.

### Informed consent

All 19 volunteers attending the CODV training programme were sent written information regarding the evaluation of the project prior to the first training session. The information sheet explained the process for data collection; completing the reflective diary and its function within the evaluation. During the first session the Researcher (KN) gave further verbal explanation of what would be involved should the volunteers agree to participate in the research. Written informed consent was obtained from all willing participants. Volunteers were assured that their volunteer role would not be affected by their decision to participate or not participate in the research, and that they could withdraw from the research evaluation at any time.

### Data collection

The participants’ reflections of the phenomenon under study were captured through the use of weekly reflective diaries. Both verbal and written guidance was provided explaining the process of reflection and a basic framework was also provided to direct the participants in the completion of the diaries (See Table 2). Following each training session, the volunteers were asked to reflect on their perspectives of the phenomenon and their learning within the context of each session and to submit their diaries before or at the next training session. This gave the volunteers time to reflect on each session and also helped to counter for degradation of recall [9]. With the permission of participants the reflective diaries were also reviewed by the volunteer co-ordinator weekly, to identify any emergent issues raised.

**Table 2:** Participant Guidance on the completion of the reflective diaries

Specific reflective diary sheet
This is to record a specific or important event
Name_______________________ Date _______________
Event to be reflected on: _______________________________________
Description- What happened?
Feelings- What were you thinking & feeling?
Evaluation- What was good & bad about the experience?
Analysis- What sense can you make out of the situation?
Conclusion- What else could you have done?

### Ethical considerations

Relevant ethical approvals for the study were obtained from the North West Wales NHS Research Ethics Committee (REC reference WN/12/0040) prior to recruitment of the volunteers.

### Data analysis

Data from the reflective diaries were analysed thematically [10]. Each entry was read through twice as a minimum by the researcher and key phrases were highlighted within the text. Initial thoughts and interpretations were recorded in the text margins from which initial themes were subsequently generated. To promote the contextual validity and consistency of these initial themes, a random selection of 10 % of the individual diary entries (I am assuming that this was the case?) was also independently analysed by a second researcher who had not been involved in the initial data analysis (HC). A third researcher (AG) then revisited all of the original reflective diaries to review the data in terms of the initial themes that had been generated to ensure they captured the richness of the phenomenon under study [10].

## Results & discussion

- Nineteen volunteers completed the training programme.
- Seventeen volunteers continued to Phase 2 of the pilot (implementation of the service).
- All 19 participants completed the reflective diaries:

Ten completed the reflective diary by hand and the remainder electronically. Table 3 illustrates key demographic information for these 19 participants.

**Table 3:** Participant demographics

Variable		% (*n*)
Age: range 18–79; median age 62		
Gender	% Female	79 % (*n* = 15) 4:1 female to male ratio
Ethnicity	White/British	84 % (*n* = 16)
Employment status	Retired	47 % (*n* = 8)
Marital status	Married	47 % (*n* = 8)
Religion	Christian	84 % (*n* = 16)

- From a potential 228 diary entries over the 12 week training programme, 178 diary entries were submitted (78 %). Where potential entries were not recorded, this was predominately due to participants missing training sessions due to competing priorities (for example, holidays and family duties, work or sickness). Diary entries varied in quantity from half an A4 page at week one, progressing to four A4 pages for a single session at week nine of the training for one participant (ID 07). However, for the majority of participants the depth and richness of entries developed over time. A number of participants did not appear to be able to engage with the reflective process and used the diary entry to list the content of sessions rather than their personal thoughts and feelings (*n* = 4). Whilst journal entries from these 4 participants were not specifically excluded from the analysis, their limited emotional and personal content meant that they contributed little to the identification and interpretation of the themes generated. The initial themes identified by researcher 1 (KN) were broadly independently confirmed by researcher 2 (HC) in the 10 % sample of diary entries reviewed. Where minor differences of interpretation existed these were reconciled through discussion and further joint analysis, where appropriate. The third researcher (AG) revisited the original transcripts to ensure that the themes identified best represented the phenomenon as described by the participants. This final stage of the analysis provided an opportunity for further elaboration and integration into the higher order themes presented: Dying alone and the importance of being present
- Personal loss and the reconstruction of meaning
- Self-awareness and personal growth
- Self-preservation and coping strategies
- Group unity/cohesion

### Dying alone and the importance of being present

Providing a presence to dying patients and their families and ensuring that they did not face the dying process alone was cited as a core motivating factor for the volunteers’ involvement and was a repeated theme throughout the diary entries.

“I was thinking about sitting at the bedside of a dying person, one to one, and wondering can I give the gift of my presence to take the loneliness in dying away what a precious thing it would be to achieve”(ID 01)

However the concept of presence was more complex for the participants in this study than simply being at the bedside. Deeper understanding of the issue appeared to develop as the training progressed, with participants’ reflections moving from the more practical issues regarding how they might “communicate” or demonstrate this presence to the deeper spiritual and psychological components of what it means to provide a presence with another.

One participant reflected that she would need to, “forget all (her) concerns and concentrate on the conversations” (ID 11) whilst another described this process as an empathic shift in focus, from their own world view to that of the patient or relative:

“The project is not about us but about them, and I think as individuals we will have to utilise the humble side of our personalities once we start the volunteer work project.” (ID 11)

Interestingly, the theme of preventing patients dying alone was revised during the course of the training, with the realisation that the role could not necessarily provide presence at the moment of death. Reflections revealed a growing awareness that their ongoing role was to provide a period of presence and companionship to the dying patient, even if they died at a later time;

“I would like for them to feel that someone cares for them and loves them and that they can pass more peacefully at the end”. (ID 07)

Reflecting specifically on the content of a particular session, in which images of death and dying had been shared with the group, one participant focused on the sense of “uncertainty” in dying and the volunteer’s role in supporting patients and families with this ambiguity (ID02). However, the concept of “support” was questioned by a further participant, reflecting on the challenge of being present with people and accepting “where” they are without the need to direct or give advice. They reflected on the distinction in this form of support from other supportive and caring roles they had provided in the past: “I think it will be a continuous process for me to cultivate presence in the dying situation…” (ID 07). Another reflected that demonstrating a spiritual presence often transcends language and that perhaps the most appropriate expression of spirituality is “silence” (ID 11).

The following themes specifically illustrate ways in which the training had contributed to the participants’ learning and personal development over the course of the Education and Training Programme.

### Personal loss and the reconstruction of meaning

In addition to the desire to prevent people from dying alone, personal bereavements and losses appeared to be another significant motivating influence for participants to initially engage in the training and in their onward recruitment to the role.

“I have very vivid memories of when my sister in law ‘my friend’ was in a hospice for 2 weeks before she died. I realise now that this has prompted me to get involved with this project and become a volunteer” (ID 06)

In developing the education programme, considerable time had been allocated for participants to share their experiences and their own accounts of loss and bereavement as a means to facilitate the reflective process within the training and through the reflective diaries. It also served to highlight any unresolved issues pertaining to their own grief that may impact on the support they could give to patients and families, and to promote their own self-care.

The theme of personal loss was evident throughout the diary entries, with participants revisiting their own experiences in the light of new learning received in the training sessions. For some participants, their previous experiences had been positive and there was an altruistic desire to emulate the care that their loved ones had received. Whilst others had had a negative personal experience and were motivated to improve the experience of death and dying for others. This theme was further expanded after the training session in which participants were asked to define what constitutes a “good death”, leading them to “unpack” the specific components of a person’s death, and explore what it is that can be helpful or beneficial to both the patient and the family at this time. For some, (*n* = 5) this led to personal reflection and a redefining of their own experiences, assessing what had contributed to their feelings that the deaths that they had experienced had been “good” or otherwise. One participant reflected on her growing awareness of how important the presence of others had been to support her, and she surmises that this had defined her own experience of bereavement:

“I realise that I have been very lucky. I loved my father, mother and brother dearly but could not have wished their life prolonged unless there was a miracle… I know too that I have never felt alone, as there have always been family with me and for me.” (ID 13)

The process of revisiting and exploring their own experiences resulted in new perceptions and a revision of meaning.

“My cousin was only 41 when he died of Myeloma… there was a lot of sadness and anxiety around the whole situation,… but there doesn’t need to be stress if you remain present to your feelings and emotions…It’s made me think that I need to live each day to the fullest.” (ID 07)

One participant reflected that her own experience of working for many years in a hospital had perhaps “desensitised” her, leading her to question her sense of “normality”. (ID 06) Whilst another participant who previously believed that her experiences would be helpful to the role, now reflected that they could potentially inhibit her volunteering (ID 08).

For some participants, the training programme revealed a wider world, with different experiences of dying and loss than they had known. One participant reflected;

“I am obviously very naïve - but I can’t imagine any family fighting in the presence of someone who is dying”. (ID 12)

In this way, the training programme and process of reflection provided participants with a revised and broader context to explore their personal experiences of bereavement and loss allowing them to gain new understanding and develop new meaning.

### Self-awareness and personal growth

The Education and Training programme aimed to hone the life skills of the participants in accordance with international experiences of such volunteer programmes, mainly in the Netherlands, Italy and Germany [11]. It did not look to professionalise the volunteers, which would have been contrary to the broader objectives of the project in creating a “community presence” at the bedside. Deeper learning in the context of this study was defined as the ability to reflect on the content of the sessions and develop a sense of self-awareness and insight which could then be applied to their role as a Volunteer. In this way, the participants’ reflections revealed a growing sense of awareness that their learning was less about knowledge and more about their emotional, spiritual, social and philosophical development. “I have a lot to learn and it is not just about knowing, but about handling my feelings and reactions” (ID 13)

“After the training today I feel a lot more calm and competent about the whole idea, I really feel settled and kind of awe inspired” (ID 10)

Diary entries revealed participants’ questioning how they might manage their own experiences, with the recognition of the potential impact on their behaviour and ability to provide support:

“The more too, I realised the emotions that could/would be present at the bedside and in me… It will be a struggle to hold the responsibility and the feelings- and a desire to help.” (ID 07)

Moreover, the participants also shared their growing awareness on the impact that the training was having on their personal lives and beliefs, leading them to question and challenge their way of being, particularly in relation to their relationships with others and their individual styles of communicating;

“… Is it because I find it hard to talk about my own personal problems so that I don’t always symphonize (sic) with people who want to talk about their problems?” (ID 01)

Two participants reflected on the influence the training had on their own life philosophy and sense of mortality;

“…I’m hoping that my volunteering role will help me to value my time and talents even more while I am on this earth- I don’t want to be on my death bed with “the music still in me” as they say”. (ID 07)

“I believe that by doing this type of volunteer work, one ponders their (sic) own existence and being.”(ID 14)

Whilst others reflected;

“I think the training will help me to grow as a person and learn things about myself”. (ID 06)

“Every week on this course I have been motivated to think about things on a deeper level” (ID 11)

### Self-preservation and coping strategies

A growing personal awareness prompted the participants to explore ways that they might cope with the impact of their future role.

“I do need to prioritise my own well-being so that I stay well” (ID 02)

Participants began to develop their own strategies to separate the emotional content of the training sessions with their home and personal lives and this then formulated their thinking of how they might cope in their ongoing role as a volunteer.

“I have found that taking some time out after the training and just getting my head around things enables me to appreciate the training and understand, but also not let it have a detrimental impact on my life”(ID 03)

“I am thinking what could be my bridge, the point where I enter a volunteer role and the exit point where I come back to my life” (ID 05)

In one session, the group was shown images of people who were near to death to illustrate some of the physical changes that may occur during the dying process, aiming to prepare the group for some of the visual and potentially distressing symptoms they may witness in their role. The subsequent diary entries revealed the impact of this exercise, with a range of responses from those who found it sad or distressing (*n* = 2) to those who did not appear too shocked or disturbed by the images (*n* = 3) and the remainder not commenting on this particular exercise within their diary entries. This exercise appeared to be particularly powerful in provoking further thinking around how they might cope in their volunteering role and the need to find an appropriate outlet for the human distress and impact of loss that they might experience. Furthermore, participants reflected on their personal resonance with the images and began to identify the individual meaning that the loss had for them:

“I felt moved and sad because I could see that some of these patients had suffered. However, I consoled myself by the thought that their suffering was now over and they were at peace. This is something I will remember on leaving a patient who has died.”(ID 02)

“The graphic photographs did not shock or upset me at all…I was very moved by the pictures of the couples, their love was palpable to me. Human feelings are very important to me, much more than what people look like.”(ID 06)

In this way, participants used their reflections to project forward and prepare for their Volunteering role, using the sessions to develop a framework of approach and personal understanding of how they might behave and cope;

“My aim will be to tune into the emotions without being overtaken by them. I want to be watchful and learn to protect myself without becoming depersonalised.” (ID 13)

“The uneasy side of it is controlling our own emotions, and finding ‘the place’ as and when to switch off” (ID 04)

For some participants (*n* = 8) the sessions which addressed the practical aspects of the role, such as how to enter and leave the room after an episode of care, were the most helpful in allowing them to explore and experiment how they might behave in the role. However one participant (ID 13) noted that this might, again; detract from the “naturalness” of the encounter and the uniqueness of the volunteer role, distinct from the clinical care provided by professionals.

All participants reflected that the training programme had been comprehensive in preparing them for their volunteering role, although responses varied from an excited

“I can’t wait to start, bring it on” (ID 12)

To a more considered;

“I am anxious, insecure, but excited.... and hopeful” (ID 13)

Four participants noted that the planned visit to the ward areas with Specialist Palliative Care Team members was eagerly anticipated and a real opportunity to contextualise their learning.

### Unity/cohesion

All participants reflected that the group in and of itself, was cohesive and facilitative;

“I was very aware of how easily we spoke in the group about very precious emotional memories. We have become a very supportive, comfortable group” (ID 13).

“At this time and throughout the day I was very aware of the supportive cohesion and trust within the group” (ID 11).

In the development of the Education and Training Programme it was acknowledged that the group environment would have a significant influence in enabling participants to share and reflect. This was further strengthened by the negotiated group contract setting boundaries and an agreed framework for the group to feel safe and supported. However, one participant highlighted a lack of adherence to the agreed rules of the group contract referring to an issue when a group member had been spoken over by two other participants (ID 01).

Some participants (*n* = 3) who took a less active role in group feedback, reflected on their discomfort sharing and discussing issues with the wider group. However for most, this appeared to lessen with time and familiarity and one participant later reflected on her “frustration” that she was not sharing her views more openly leading her to reflect on the need to increase her personal confidence and self-belief. Interestingly, following a subsequent session the same participant reflected on her growth;

“I feel proud that I contributed my own experiences of my grandmother’s death this week, I actually surprised myself as normally I am a very private person” (ID 10).

As the training progressed and the solidarity of the group grew, the participants’ reflections reveal a developed understanding of the potential contribution the service could make. Furthermore, their increasing confidence is reflected not only in their ability to share in-group discussions, but also with an increased self-belief in the validity of their own opinions and experiences.

“I cannot wait for the further discussions we’ll have as a group because we all bring a unique vantage point” (ID 14)

However, the reflective diaries were also used by some participants to explore their frustrations or disagreements with other participants on the training course. On two occasions there appears to be some personality differences and in another an opposing view or opinion. The diaries appeared to enable participants to share and explore these feelings in a safe and non-confrontational environment.

“I found one member of the group a bit dogmatic in regard to religion and morality, but I was very pleased with myself for not challenging [this person’s] views too much as I don’t feel it is appropriate trying to do in the context of the group.” (ID 06)

## Discussion

The data contained within the diaries provided a valuable insight into the experiences of the volunteers throughout the duration of the training programme. This enabled the volunteers’ journey from novice to potential CODV to be explored.

In line with previous findings by McKee et al. [12], a consistent and core motivating factor in the decision of participants to join the Care of the Dying Volunteer Programme was a strongly held belief in the importance of preventing patients from dying alone. Undoubtedly, in Western societies the concepts of “dying alone” and “bad death” are often conflated [13]. However, as the training progressed, the reflective diaries revealed a subtle but important shift in these volunteers’ perspective away from a focus on the actual moment of death of the patient, to a broader sense of ‘being there’, and ‘journeying with’ the patient during the last phase of life.

The experience of personal bereavements and losses was another significant motivating influence for participants to both engage in the training and also in their onward recruitment to the role. This finding concurs with previous research exploring motivations to volunteer for end of life care roles, predominately in the hospice environment [14–17]. However the Training programme, together with the completion of the diaries also enabled participants to revisit their own experience of loss and bereavements and for many participants, the data indicated a revision of understanding or perception.

Neimeyer [18] identifies this reconstruction as a common, though often painful and protracted, process in the aftermath of a significant loss where the bereaved search to make sense and meaning from their experience and integrate the loss into their adjusted self-constructed narrative. The training programme and subsequent reflections, allowed for a further reassessment of these experiences often leading to the new sense of meaning or “reframing” as described by Neimeyer for more acute phases of loss.

Self-awareness was considered to be a key attribute for the role as a Care of the Dying Volunteer, ensuring that participants were able to recognise their own emotional responses distinct from those of the patient or family member they were supporting. The diary data evidenced a developing self -awareness and personal growth for the majority of participants, with an increasing recognition of the connection between personal experiences and the application of the learning from the training sessions. Boud [19] advocates that effective learning is always grounded in prior experience and therefore new learning must take that experience into account. He states that an individual’s past experiences profoundly affect their perceptions of what does and does not count as important; acting as a way to sensitise a person to some features of the world and blind to others. In this way, the training programme and process of reflection provided participants with a revised and broader context to explore their personal experiences of bereavement and loss affording the potential to gain new understanding and develop new meaning.

This theme is also reflected in the findings of a recent study by Ferreira et al. [20] that illustrated that volunteerism has positive impacts on both learning and knowledge in addition to providing countless opportunities for personal growth. Boud [19] develops this theory further, stating that reflection is an essential component of experience-based learning, in which the process of reflection in itself is an experience with the potential for new perceptions, thoughts and feelings. In essence, it offers an opportunity for participants to alter their world view, and provides a vehicle for participants to develop, and grow beyond the specific learning objectives of the training programme. This concept of experience-based learning, whereby the learning involves a holistic approach in which the learner actively constructs their own experience is perhaps best defined by Kolb:

“Learning is the process whereby knowledge is created through the transformation of experience” [21] (p. 38).

The ability to reflect was also identified as an essential requirement of the ongoing volunteering role and there was a mandatory requirement for all volunteers to attend a monthly group supervision session in which they would be required to reflect on episodes of care they had provided to patients and families. Volunteers were required to Present salient points and to reflect on both the cognitive and emotional impact, which promoted opportunities for both personal and group learning. Regular group supervision also provided ongoing support allowing volunteers the time and space to reflect on their experiences in a supportive environment, and this was identified as an essential coping strategy to maintain the emotional health and well-being of the volunteering team. Reflective practice is identified as a key component in promoting resilience for all those working in end of life care and in reducing the risk of burn out or compassion fatigue [22], and this recognition of the benefits afforded by the mutuality and shared experience of the group reflect the findings of a study conducted by Claxton-Oldfield et al. [23] who detailed a variety of methods employed by volunteers to prevent burnout.

This supports the premise that it is not possible to provide support for others without the ability to care for oneself [24, 25] and concurs with Showalter [26], who identified that professional caregivers must learn to make a clear distinction and balance between their personal and professional lives in order to sustain their sense of emotional and physical wellbeing, similarly volunteers need to find this sense of balance and boundary.

In addition to this formalised peer support, the diary data revealed recognition of the importance of developing individual strategies to cope with the consequences of the role. This theme emerged at different points throughout the training programme, but particularly after the sessions that focussed on the dying process, prompting the participants to reflect both on the impact of the session, whilst also acknowledging the challenge of managing their emotions in the role of a volunteer.

The diary data indicated a strong sense of unity and cohesion within the group and this was extended into the ongoing monthly supervision sessions, which offered an opportunity for the volunteers to share their experiences of providing support. This supports the findings of a study by Costa et al. [27], who report that the experience of sharing in itself facilitates a sense of community. The theme of group cohesion was repeated throughout the diary entries and the importance of the safety of the learning environment appeared to be a key component of the training programme. Death and dying are clearly very emotive and sensitive issues, which are often culturally, or religiously embedded in beliefs and understanding. Therefore the safety of the group and environment, were essential in creating an opportunity for participants to reflect and explore their personal resonance with the issues presented. However the individual reflection provided by the completion of the diary appeared to enable many participants to explore issues at a deeper level, including their relationships within the group and the impact on their ongoing growth and development.

Clearly, reflective diaries are only one method that can be used to facilitate the process of reflection in a learning environment; other methods include story-telling, creative writing, critical incident reporting and other experiential exercises and it is important to note that reflective diaries may not offer an effective method for all learners [7]. In this study, despite the evidence that for many emotional and personal reflection was a skill that developed over the period of the study, more guidance and ongoing support in engaging effectively with the reflective process may have potentiated greater involvement in the four cases where this remained limited. Identifying innovative ways to ‘teach’ reflection as part of the training, providing ongoing feedback in response to entries made and using a variety of approaches (for example the use of ongoing verbal and written feedback to diary entries, taking into account individual learning styles) may have provided valuable support to those who found such activity challenging. It is important to remember also that the written format of such reflective diaries may prove challenging for other reasons. For example, the literacy skills of the individuals completing the diaries are also likely to be pertinent to the free expression of thoughts and feelings. An alternative option may be to use an audio or electronic diary as a means of recording.

In addition, it should be noted that within this study the decision was taken that the Training Lead should also review the diaries on a sessional basis to facilitate the delivery of a more responsive and student-centred programme. However, this may have potentially influenced the participants’ openness and level of disclosure about the training delivered and received.

Furthermore, the findings in this study are based on a relatively small sample from a single locality in the acute setting of a hospital. Generalised assumptions regarding specific end of life care training in other care settings or with other groups of participants or healthcare professionals are precluded.

## Conclusions

In this study, the use of reflective diaries provided the opportunity to examine the lived experience of a volunteer training programme to support dying patients in the acute hospital setting. The diaries facilitated an open dialogue between the volunteers, training leads and the researcher, and offered the opportunity to explore the volunteers’ understanding of the course content together with an account of their learning processes on a sessional basis.

The diaries offered an inexpensive and simple medium to promote the important and ongoing process of reflection, and for many this skill developed over the duration of the training programme and was adopted as part of their Volunteering practice and ongoing peer supervision. The diary entries revealed an important shift in focus over time from an initial preoccupation with previous experiences of death and dying to looking ahead to contextualise their learning, reflecting on how they might cope and function in their ongoing role. We believe that this is illustrative of their personal growth through exposure to the learning and reflective experience.

The volunteers’ reflections have been a driving influence in the revision and development of the education and training programme, which will now be used to train a further cohort of participants. For example, whilst the pilot reinforced the value of reflection, it also highlighted that the skill of reflection is not a given. Not everyone was able to ‘connect’ fully with the completion of reflective diaries and in order to maximise the potential for full involvement in this element of the process, further guidance, encouragement and support have been built into the training and ongoing supervision of a new cohort of volunteers.

Undoubtedly, further work is required to establish the effectiveness of the reflective diary in end of life care education for volunteers and also the applicability of the training methods for other groups and settings. A longitudinal study design which offers the opportunity to track participant’s development through training and implementation may provide more comprehensive evaluation of the reflective diary as a means to promote enhanced practice and quality in care provision.

## Abbreviation

CODV: care of the dying volunteer
